# Calcified cerebral toxoplasmosis associated with recurrent perilesional edema causing neurological manifestations in an HIV-infected individual: case report with a decade-long follow-up

**DOI:** 10.1590/S1678-9946202466015

**Published:** 2024-03-18

**Authors:** Flávia Carolina Soares Bonato, René Leandro Magalhães Rivero, Hector Hugo Garcia, José Ernesto Vidal

**Affiliations:** 1 Instituto de Infectologia Emílio Ribas, Departamento de Doenças Infecciosas, São Paulo, São Paulo, Brazil; 2 Instituto de Infectologia Emílio Ribas, Divisão de Apoio ao Diagnóstico e Terapêutica, Setor de Radiologia, São Paulo, São Paulo, Brazil; 3 Universidad Peruana Cayetano Heredia, Centro de Salud Global, Lima, Peru; 4 Instituto de Ciencias Neurológicas, Unidad de Cisticercosis, Lima, Peru; 5 Instituto de Infectologia Emílio Ribas, Departamento de Neurologia, São Paulo, São Paulo, Brazil; 6 Universidade de São Paulo, Faculdade de Medicina, Hospital das Clínicas, Divisão de Moléstias Infecciosas, São Paulo, São Paulo, Brazil; 7 Universidade de São Paulo, Faculdade de Medicina, Instituto de Medicina Tropical de São Paulo (LIM-49), São Paulo, São Paulo, Brazil

**Keywords:** Cerebral toxoplasmosis, Pathologic calcification, Edema, Neuroimaging, HIV

## Abstract

Four cases of people living with HIV/AIDS (PLWHA) with calcified cerebral toxoplasmosis associated with perilesional edema causing a single episode of neurological manifestations have recently been reported. Here, we describe the first detailed description of perilesional edema associated with calcified cerebral toxoplasmosis causing three episodes of neurological manifestations in a PLWHA, including seizures in two of them. These recurrences occurred over approximately a decade. Throughout this period, the patient showed immunological and virological control of the HIV infection, while using antiretroviral therapy regularly. This case broadens the spectrum of an emerging presentation of calcified cerebral toxoplasmosis, mimicking a well-described finding of neurocysticercosis in immunocompetent hosts.

## INTRODUCTION

Cerebral toxoplasmosis was described at the beginning of the AIDS epidemic^1^ and has become one of the most frequent opportunistic infections and the most common cause of focal brain lesions in people living with HIV/AIDS (PLWHA).

The introduction of combined antiretroviral therapy (cART) has drastically reduced the frequency of cerebral toxoplasmosis^2,3^. However, this opportunistic infection continues to be an important cause of hospital admission and mortality in PLWHA in low- and middle-income countries^4,5^.

The main neuropathological, clinical, and radiological features of cerebral toxoplasmosis, whether or not treated with anti-*Toxoplasma gondii* therapy, are well described^1,6^, and specific treatment results in high survival rates^7^.

Given the clinical evolution of cerebral toxoplasmosis, once the lesion resolution on neuroimaging and clinical improvement are obtained, little attention was paid to subsequent follow-up. On the other hand, a recent study showed that contrast-enhancing cerebral toxoplasmosis lesions observed on magnetic resonance imaging (MRI) scans can persist for months to years after anti-*Toxoplasma* therapy and cART, despite clinical resolution and successful immune reconstitution^8^. Thus, complete elimination of the lesions is not always achieved. In this scenario, there is little information on the long-term evolution of treated cerebral toxoplasmosis, including residual calcified brain lesions. Recently, four PLWHA with calcified cerebral toxoplasmosis and a single episode of perilesional edema associated with neurological manifestations were described^9^. Prior to this, calcified brain lesions associated with perilesional edema and neurological manifestations were described in individuals with neurocysticercosis, usually immunocompetent^10,11^.

Here, we present a case of calcified cerebral toxoplasmosis associated with three episodes of perilesional edema causing seizures in an HIV-infected patient, showing its clinical and radiological evolution over a decade.

## CASE REPORT

**Figure 1:**
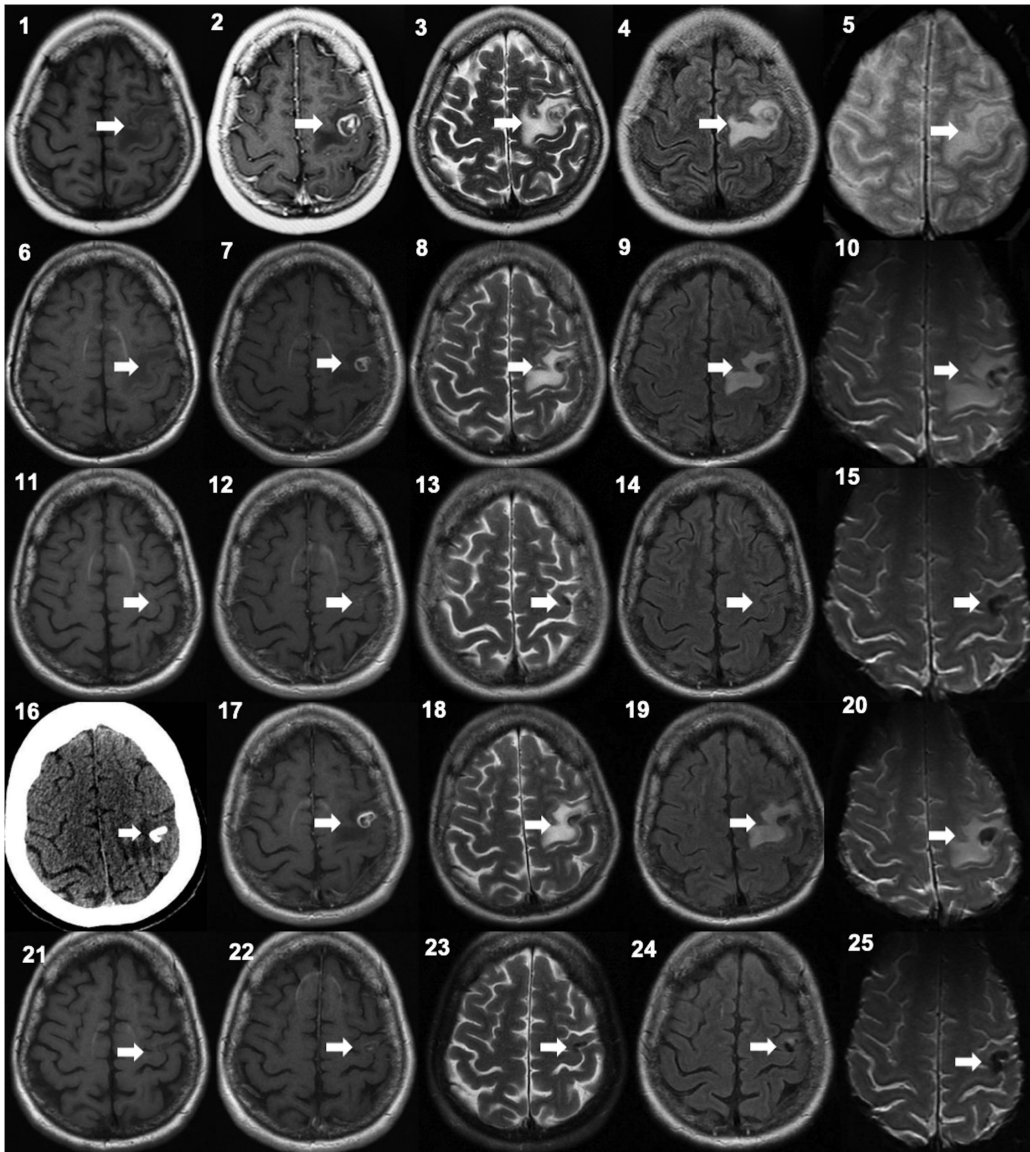
Neuroimaging showing the findings of the initial diagnosis of cerebral toxoplasmosis and the first and third subsequent episodes of perilesional edema and enhancement around a calcification secondary to this opportunistic disease. In the first row: initial MRI (10/2010) with the lesion located in the precentral gyrus. (1) T1-weighted image, before contrast administration and (2) post-gadolinium image showing ring enhancement with an eccentric nodule in this lesion; in (3) and (4), T2 and FLAIR images, respectively, showing associated edema in the surrounding white matter. In (5), T2* image (GRE), with no signs of calcification or hemorrhage in this lesion; In the second row: MRI of the first episode of perilesional edema associated with calcification (11/2015). T1-weighted image showing the previous lesion with a smaller size, (6) before and (7) after gadolinium; in (8) and (9), T2 and FLAIR images, respectively, showing associated edema in the surrounding white matter. In (10), T2* image (GRE), some low signal areas can be seen, which may represent early calcium deposits; In the third row: following MRI (03/2016) showing resolution of the first episode of perilesional edema associated with calcification. T1-weighted images, (11) before and (12) after gadolinium, showing significant regression of the lesion enhancement and T2 (13) and FLAIR (14) images without edema. In (15), T2* image (GRE), better definition of the pronounced low signal areas, suggesting intensification of the calcium deposits in the lesion site; In the fourth row: CT and MRI (12/2021) of the last episode of perilesional edema associated with calcification. (16) CT showing calcification in lesion site with surrounding edema and (17) MRI T1-weighted post-gadolinium image with lesion enhancement, as well as increased perilesional edema in T2 (18) and FLAIR (19) images. In (20), T2* image (GRE), increasing of the pronounced low signal areas, compatible with the calcification showed in the concomitant CT scan (16); In the fifth row: subsequent MRI (04/2022) of the third episode of perilesional edema associated with calcification. T1-weighted images, (21) before and (22) after gadolinium, showing resolution of the inflammatory activity with minimal enhancement and the disappearance of the edema in the T2 (23) and FLAIR (24) images. In (25), T2* image (GRE) showing persistence of pronounced low signal areas, compatible with calcification (16).

A 52-year-old woman was admitted to our hospital emergency room (ER) with a history of seven self-limited focal seizures in the right upper limb and ipsilateral hemiface in the last 12 hours. Her medical history consisted of advanced HIV disease and a well-documented episode of treated cerebral toxoplasmosis presenting right hemiparesis 11 years ago (Figure 1, 1–5). In the weeks following the cerebral toxoplasmosis diagnosis, the patient presented adequate immune reconstitution and undetectable HIV-1 viral load in use of cART. Between the initial diagnosis of cerebral toxoplasmosis and the current evaluation at the ER, the patient had two other episodes of neurological symptoms, always under regular use of cART with cerebral MRI findings compatible with calcification associated with perilesional edema and enhancement after gadolinium administration. In the first episode, six years ago, the patient was hospitalized for focal seizures and received carbamazepine (Figure 1, 6–10). Four months after discharge, an MRI showed resolution of the perilesional edema associated with calcification (Figure 1, 11–15). In the second episode, one year ago, she was evaluated on an outpatient unit because of self-limiting headache episodes that did not require symptomatic medication (Figure not shown).

**Figure 2:**
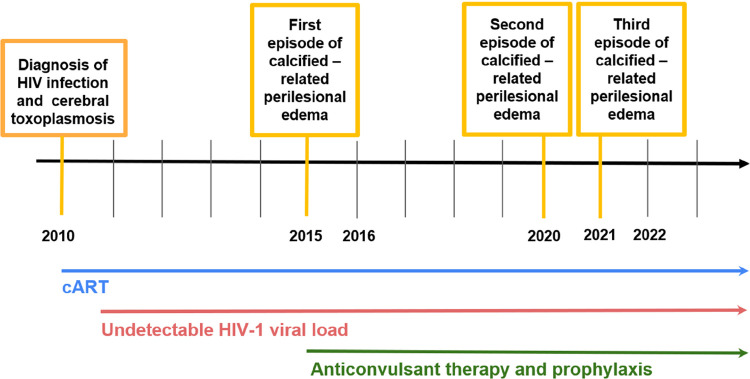
Timeline of the case report. cART: combined antiretroviral therapy.

At the ER, the patient had two additional focal seizures in the right upper limb. On neurological examination, she had discrete distal muscle weakness in the right upper limb (4+/5), reported as a sequela since the initial diagnosis of cerebral toxoplasmosis. The patient reported regular use of tenofovir disoproxil fumarate, lamivudine, dolutegravir, and lamotrigine 50 mg BID. Her CD4+ count was 2,269 (30%) cells/mm^3^and her HIV viral load was < 40 copies/mL. Non-contrasted brain computed tomography (CT) scan showed two images compatible with calcifications and a mild perilesional edema was observed around one of them (Figure 1, 16). A corresponding MRI at that time showed these calcifications and the lesion with perilesional edema was increasing after gadolinium administration (Figure 1, 17–20). The calcification site was the same as that of the initial episode of active cerebral toxoplasmosis 11 years before (Figure 1, 1–5). Cerebrospinal fluid examination was unremarkable. Serum IgG anti-*T. gondii* was positive. Serum enzyme-linked immunoelectrodifusion transfer blot for cysticercosis was negative. The electroencephalogram, performed three days after the last focal crisis, was normal. The patient received prednisone 1 mg/kg/day, omeprazole 20 mg/day, and the dose of lamotrigine was progressively increased to 100 mg BID. Two weeks later, the patient was asymptomatic and a new MRI showed marked improvement in perilesional edema and contrast enhancement (figure not shown). She was discharged with a progressive reduction in prednisone dose until discontinuation over the following six weeks, while maintaining all other medication. Three months after discharge, the patient was evaluated in our outpatient clinic, continued to be asymptomatic in regular use of medications, and a non-contrast brain CT showed resolution of the calcification-related perilesional edema, a finding confirmed by a corresponding MRI that identified minimal contrast enhancement in the calcified lesion (Figure 1, 21–25). Her CD4+ count was 2,417 (28%) cells/mm^3^ and HIV-1 viral load was < 40 copies/mL. The timeline of the clinical evolution of the patient is showed in Figure 2.

This case report was authorized by the patient and by the Research Ethics Committee and Scientific Division of the Instituto de Infectologia Emilio Ribas (Protocol Nº 23/2023).

## DISCUSSION

We report the case of a PLWHA with cerebral calcification corresponding to prior cerebral toxoplasmosis associated with recurrent perilesional edema and neurological manifestations. Previously, only four PLWHA with calcified cerebral toxoplasmosis associated with a single episode of perilesional edema causing neurological manifestations were described^9^.

**Figure 3:**
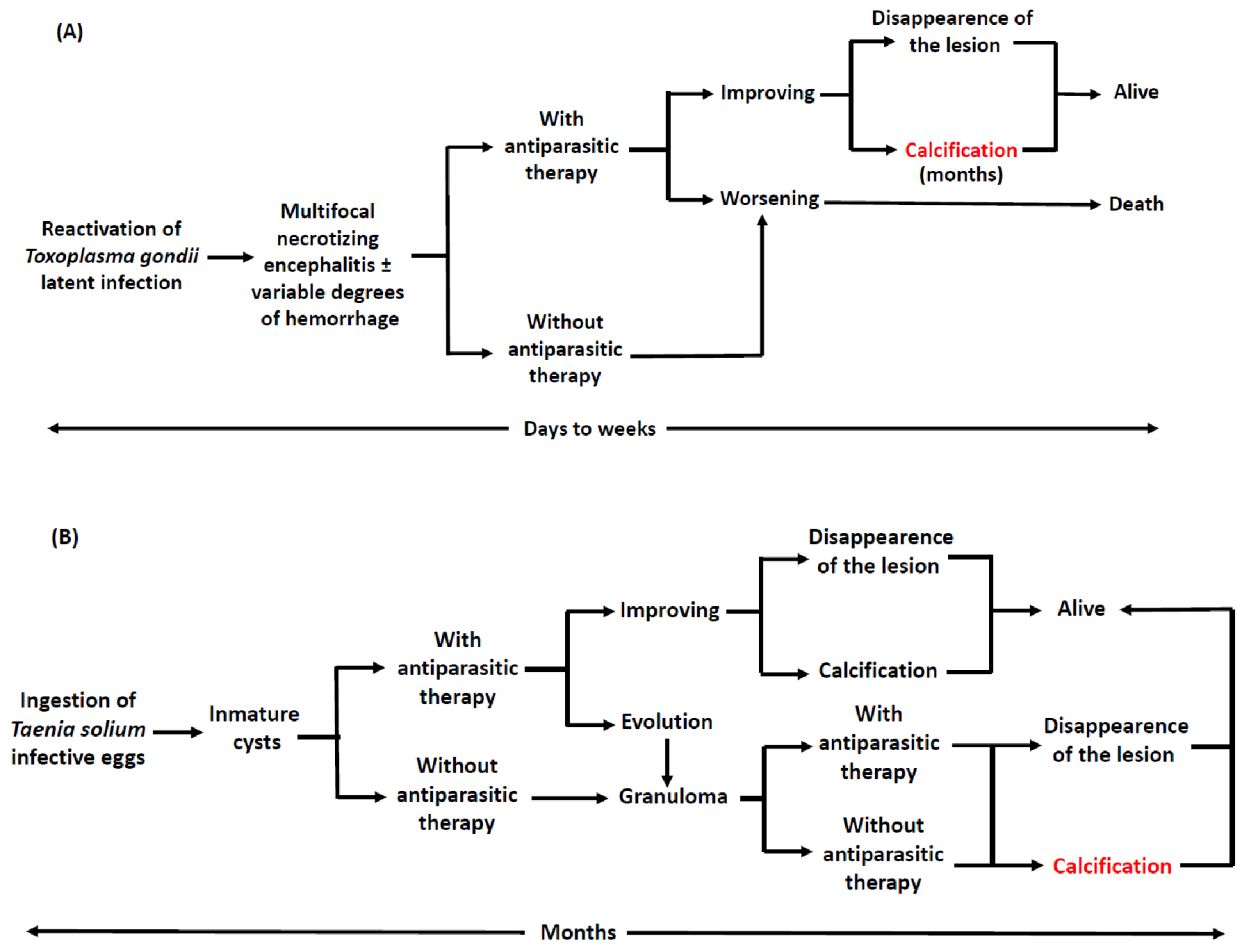
Simplified scheme of the evolution of cerebral toxoplasmosis (A) and parenchymal neurocysticercosis (B) lesions with or without antiparasitic therapy.

Parasitic diseases are a major public health problem worldwide, resulting in a significant disease burden in low- and middle-income countries^12,13^; neurocysticercosis and cerebral toxoplasmosis are the most frequent neuroparasitosis in immunocompetent individuals and PLWHA, respectively. The natural history and the impact of antiparasitic therapy also show marked differences between these two diseases, as summarized in Figure 3.

Cerebral calcification can be a common final outcome of several diseases or conditions (physiologic/age-related, dystrophic, congenital disorders/phakomatoses, infectious, vascular, neoplastic, metabolic/endocrine, inflammatory, and toxic diseases)^14,15^. The neuroradiological patterns of calcification generally differ between neurocysticercosis and cerebral toxoplasmosis. The calcified neurocysticercosis lesions are typically small, rounded, well-defined calcified nodules less than 1 cm in diameter. On the other hand, calcified cerebral toxoplasmosis commonly presents a spectrum of dot-like, or thick and “chunky,” or “brain stone” lesions^9^.

Neurocysticercosis is the most important and studied infectious disease causing cerebral calcifications in adults^11,16^. However, research on calcified neurocysticercosis is long overdue and many questions remain unsolved^17^. Congenital toxoplasmosis is the most important and studied infectious diseases causing cerebral calcifications in pediatric populations^18^. However, cerebral calcifications in acquired cerebral toxoplasmosis are poorly characterized, despite being the most common opportunistic neurological disease during almost four decades of the HIV epidemic^6^.

Some arguments may justify, at least partially, the little attention given to calcifications secondary to acquired cerebral toxoplasmosis. Firstly, the acute/subacute nature of the disease and the potential for mortality may have focused attention on hospital care for the acute management of reactivated disease. Secondly, in daily clinical practice, calcified toxoplasmic lesions are generally understood as quiescent sequelae of the disease. Finally, cerebral toxoplasmosis, like other opportunistic diseases, occurs mainly in countries with limited resources, including neuroimaging, and thus the late consequences of the disease may not have been identified in their real consequences and impact.

Classically, calcified cerebral lesions were referred to as “inactive,” but studies on calcified neurocysticercosis have changed this concept, demonstrating that the calcification process is dynamic and can contribute to the development and maintenance of neurological manifestations, particularly seizures^16,17^. Perilesional edema, symptomatic or asymptomatic, is a well-characterized finding in cases of calcified neurocysticercosis^11^. Late structural abnormalities secondary to neurological infections, including neurocysticercosis and cerebral toxoplasmosis, hold epileptogenic potential and consequently lead to perilesional edema. However, the pathophysiology of calcified cerebral lesions associated with perilesional edema is unknown. Currently, it is still unclear whether peri-calcification edema observed in patients with seizures result from an immunological mechanism (recognition of parasite antigens in the calcified matrix) or whether edema is secondary to seizure-induced blood-brain barrier disruption. The persistent contrast enhancement in the periphery of some calcified lesions, the peri-calcification edema in certain patients without seizures, and the chronology of the clinical-radiological findings observed in some cases, suggest an immunological origin^9^. This rationale has been proposed for neurocysticercosis, but a similar mechanism is likely in cerebral toxoplasmosis. There is no explanation for the fact that only some calcified lesions in neurocysticercosis are associated with edema and that only some of them cause seizure activity or other clinical manifestations^19^. We recently reported a series of four cases of HIV-related toxoplasmosis with current virological and immunological control of HIV infection, which presented perilesional edema around their cerebral calcifications^9^, suggesting that calcified cerebral toxoplasmosis may cause perilesional edema and symptoms in PLWHA, similar to neurocysticercosis.

The current state of knowledge about calcified neurocysticercosis is the result of clinical-epidemiological (e.g., cross-sectional and cohort) and experimental (e.g., animal models and histopathology) studies. Similar studies on calcified cerebral toxoplasmosis are needed to better know and understand its late consequences and optimize its management. In the meantime, timely recognition of calcified cerebral toxoplasmosis associated with perilesional edema can avoid unnecessary antiparasitic therapy or more invasive procedures^9^. Furthermore, similar to calcified neurocysticercosis with perilesional edema, in which there is no proven therapy, symptomatic treatment may be useful^11^. We propose that corticosteroids can be evaluated on a case-by-case basis, considering that abrupt withdrawal may induce perilesional edema, as described in neurocysticercosis^20^. We followed these considerations in the management of the present case.

## CONCLUSION

In conclusion, this case demonstrates that individuals with calcified cerebral toxoplasmosis associated with perilesional edema can present recurrences characterized by clinical and radiological manifestations. This presentation is similar to that reported in neurocysticercosis. Anticonvulsant medication seems to be useful as in other cases of secondary epilepsy, but the benefit of corticosteroids is still controversial.
